# Spatio-Temporal Expression Pattern of Frizzled Receptors after Contusive Spinal Cord Injury in Adult Rats

**DOI:** 10.1371/journal.pone.0050793

**Published:** 2012-12-10

**Authors:** Pau Gonzalez, Carmen Maria Fernandez-Martos, Carlos Gonzalez-Fernandez, Ernest Arenas, Francisco Javier Rodriguez

**Affiliations:** 1 Molecular Neurology Laboratory, Hospital Nacional de Paraplejicos, Toledo, Spain; 2 Molecular Neurobiology Unit, Department of Medical Biochemistry and Biophysics, Karolinska Institute, Stockholm, Sweden; University of Pittsburgh, United States of America

## Abstract

**Background:**

Wnt proteins are a large family of molecules that are critically involved in multiple central nervous system (CNS) developmental processes. Experimental evidences suggest a role for this family of proteins in many CNS disorders, including spinal cord injury (SCI), which is a major neuropathology owing to its high prevalence and chronic sensorimotor functional sequelae. Interestingly, most Wnt proteins and their inhibitors are expressed in the uninjured spinal cord, and their temporal expression patterns are dramatically altered after injury. However, little is known regarding the expression of their better-known receptors, the Frizzled family, after SCI. Thus, the aim of the present study was to evaluate the expression of Frizzled receptors in the damaged spinal cord.

**Findings:**

Based on the evidence that Wnts are expressed in the spinal cord and are transcriptionally regulated by SCI in adulthood, we analysed the spatio-temporal mRNA and protein expression patterns of Frizzled receptors after contusive SCI using quantitative RT-PCR and single and double immunohistochemistry, respectively. Our results show that almost all of the 10 known Frizzled receptors were expressed in specific spatial patterns in the uninjured spinal cords. Moreover, the Frizzled mRNAs and proteins were expressed after SCI, although their expression patterns were altered during the temporal progression of SCI. Finally, analysis of cellular Frizzled 5 expression pattern by double immunohistochemistry showed that, in the uninjured spinal cord, this receptor was expressed in neurons, oligodendrocytes, astrocytes, microglia and NG2^+^ glial precursors. After injury, Frizzled 5 was not only still expressed in oligodendrocytes, astrocytes and NG2^+^ glial precursors but also in axons at all evaluated time points. Moreover, Frizzled 5 was expressed in reactive microglia/macrophages from 3 to 14 days post-injury.

**Conclusions:**

Our data suggest the involvement of Frizzled receptors in physiological spinal cord function and in the cellular and molecular events that characterise its neuropathology.

## Introduction

To date, 19 Wnt ligands belonging to the Wnt family of proteins have been described in mammals [1]. These Wnt proteins are able to activate different signalling pathways, which have classically been divided into canonical and non-canonical pathways [2]–[4]. In addition, the activation of the Wnt-dependent signalling pathways is modulated by co-receptors, such as kremen 1/2, and antagonists, such as Wnt inhibitory factor 1, dickkopf (Dkk) and secreted Frizzled-related proteins [5], [6].

Wnt ligands are able to act through the so-called non-conventional receptors, such as Ryk, receptor tyrosine kinase-like orphan receptor 1/2 and protein tyrosine kinase 7 [7], which can function as either co-receptors or autonomous receptors [8]. However, the better-known Wnt receptors are the 10 known Frizzled (Fz) receptors (Fz1-10) [8].

In the central nervous system (CNS), most of our knowledge about Fz functions comes from developmental studies. During CNS development, Fz receptors are involved in many cellular processes, such as migration, proliferation, differentiation, polarisation, axonal growth, dendritic arborisation and synapse formation, which lead to the correct regionalisation and vascularisation of different CNS anatomical areas and to the proper formation of many neural circuits [1], [9]. Notably, Fz receptors may also play important roles in CNS function under physiological conditions during adulthood [10] and, interestingly, under pathological conditions in neuropathologies such as cancer [11], [12], Parkinson disease [13], and Alzheimer disease [14]. Moreover, strategies seeking to modulate Wnt-dependent signalling pathways have proved beneficial in several experimental models of CNS disorders [14]–[23].

Spinal cord injury (SCI) is a major neuropathology, as it affects a significant proportion of the population, induces long-term disabilities and has no universally accepted treatment [24]. SCI starts with the primary injury phase, characterised by an initial damage core generally induced by the application of mechanical forces [25]. As a consequence, the secondary injury phase is induced, which, in turn, is characterised by the activation of a complex network of cellular processes, such as microglial and astroglial reactivity, leukocyte infiltration and mobilisation of neural precursors, that are tightly regulated by a wide range of molecules, such as cytokines, chemokines, growth factors and inflammation-related enzymes. Interestingly, the activation of the secondary injury phase usually leads to the massive death of neural cells and to the disruption of neural circuits that surround the primary injury core, and these cause most of the functional deficits associated with SCI [25]. However, our knowledge of the molecules that regulate the biological processes that characterise the secondary injury phase is still scarce, limiting our capacity to interact with these biological processes and, thus, to generate new potential therapeutic strategies.

Intriguingly, many of the few actually known functions exerted by the Wnt family of proteins in neuropathological conditions affect to biological processes that are usually associated to the secondary injury phase. For instance, Wnts are involved in neuron survival [14], [15], [18]–[22], [26]–[29], microglia/macrophage reactivity [30]–[35], axonal regeneration [36]–[39] and the mobilisation and differentiation of neural precursors [16], [40], [41]. Specifically in SCI, a previous study performed by our group has not only demonstrated that most Wnt ligands and their inhibitors are expressed in the lesioned spinal cord in strictly regulated gene expression patterns but also that Wnt-dependent signalling pathways are activated [42]. Moreover, recent reports have demonstrated that after SCI Wnts may exert critical functions in myelin loss [40], in the modulation of axonal regeneration [36], [38] and in the differentiation of neural precursors [40]. However, our knowledge is still far away from a complete understanding of the potential roles of the Wnt family of proteins in SCI. In this context, the expression pattern of the Fz receptors during the progression of SCI, knowledge of which is essential for ascertaining the potential roles of Wnt signalling in this neuropathology, has not been studied. Therefore, the aim of the present experimental work was to evaluate the mRNA and protein expression patterns of the known Fz receptors in the uninjured spinal cord and after contusive SCI.

## Materials and Methods

### Animals

To perform the present study, a total of 36 male Wistar rats (3 months of age, ∼300 grams) were used. Animals were housed in climate-controlled quarters with a 12 h light/dark cycle and with ready access to food and water. All experimental procedures were carried out in accordance with the European Union directives (2010/63/EU) and the NIH guidelines for the care and use of experimental animals and were approved by the Bioethics Committee at the National Hospital of Paraplegics (Toledo, Spain) (Permit numbers 51/2009 and 45/2008).

### Contusive spinal cord injury and experimental design

The contusive spinal cord lesions were performed as previously described [42], [43]. Briefly, animals were anesthetised by intraperitoneal administration of pentobarbital (40 mg/kg) and xylacine (10 mg/kg), placed on a thermal pad to maintain normothermia, and a laminectomy was performed at the level of T8. Following, the injury was induced via a controlled contusion (200 kilodynes) using an Infinite Horizon Spinal Cord Impactor (Precision Systems and Instrumentation LLC) and the overlying muscle and skin were sutured. The postoperative cares included subcutaneous injection of buprenorphine at 24 hours post-injury (hpi) (Buprex, 0.03 mg/kg) and enrofloxacine (Baytril, 2.5 mg/kg) once daily until 5 days post-injury (dpi). In addition, the lesioned animals received subcutaneous injections of saline solution for the first 5 dpi in decreasing doses from 5 ml at 24 hpi to 1 ml at 5 dpi. The bladders were emptied twice daily until the lesioned animals completely recovered normal bladder function and were daily inspected for signs of infection, dehydration or autophagia.

To establish a homogeneous group in which the variations in the mRNA and protein levels of the different Fz receptors were strictly due to temporal changes after injury, the Basso-Beattie-Bresnahan locomotor scale was performed as described previously at 1, 3, 7 and 14 dpi [44]. Only those animals with a functional score between 0 and 3 at day 1 after surgery and with similar functional improvement up to 14 dpi were included in the study (Figure S1).

To evaluate the mRNA expression and the spatio-temporal protein expression pattern of the Fz receptors using quantitative RT-PCR (RT-qPCR) and immunohistochemistry, respectively, lesioned animals were sacrificed as detailed in the corresponding subsections at 6 and 24 hpi and at 3, 7 and 14 dpi (n = 3 per time point and experimental technique). As control animals, 3 non-lesioned (NL) rats per experimental technique were used.

### mRNA isolation and RT-qPCR analysis

The animals used to quantify the mRNA expression of the different Fz receptors were anesthetised by intraperitoneal injection of pentobarbital (40 mg/kg) and perfused intra-aortically with 150 ml of heparinised saline solution. Total RNA was isolated using the RNeasy Lipid Tissue Mini Kit (Qiagen) according to the manufacturer's instructions from a 1-cm-long spinal cord fragment containing the wound epicentre. The protocol used for the synthesis and amplification of the complementary DNAs (cDNAs) as well as the relative quantification of the Fz receptor mRNAs and 18S rRNA (endogenous control) has been described in a previous report [42]. The cDNAs of the Fz receptors were amplified using specific primers, which were validated on embryonic cDNA before use (Fz6: forward 5′-TTGATGCGGAAAGGAGCATAA-3′, reverse 5′-CATCTTCCCCAGACTCCGATT -3′. Fz9: forward 5′-GTGGTTTTGACTCTCACCTGGTT-3′, reverse 5′-GCTTCGTGGCCCCACTT-3′. GenBank accession numbers: NM_001130536.1 and NM_153305.1, respectively). The primers that are not specified had previously been validated [45].

### Immunohistochemistry

The animals used to evaluate the spatio-temporal expression pattern of the Fz receptors by immunohistochemistry were anaesthetised by intraperitoneal injection of pentobarbital (40 mg/kg) and perfused intra-aortically with 150 ml of heparinised saline solution followed by 1 mg/kg of 4% paraformaldehyde. The spinal cord fragments were then immediately removed, post-fixed for 4 h in the same fixative solution, cryoprotected by immersion in 30% sucrose for 48 h, frozen in Neg-50 frozen medium (Richard-Allan Scientific) using dry CO_2_ and stored at −20°C. From each spinal cord, a 3 cm stretch (1.5 cm rostral and 1.5 cm caudal to the wound epicentre) was cut in a cryostat to obtain transverse parallel sections (30 µm), which were mounted on slides and stored at −20°C.

A set of parallel sections in each animal were used to visualise each Fz receptor by single immunohistochemistry, except for those which showed undetectable levels of mRNA expression using RT-qPCR (*Fz1*, *Fz3* and *Fz6*). The immunohistochemistry protocol used has been previously described [42]. The following primary antibodies produced in rabbit and obtained from Abcam were used: anti-Fz2 (ab75084) (1∶1000), anti-Fz4 (ab83042) (1∶2500), anti-Fz5 (ab75234) (1∶250), anti-Fz7 (ab64636) (1∶2000), anti-Fz8 (ab75235) (1∶250), anti-Fz9 (ab61430) (1∶250) and anti-Fz10 (ab83044) (1∶1000). All sections used to evaluate the expression pattern of each Fz receptor by single immunohistochemistry were processed at the same time and following the same experimental protocol.

To evaluate the cellular Fz5 expression pattern in both non-lesioned and lesioned spinal cords, double immunohistochemistry was performed in a set of parallel sections for each animal to visualise Fz5 in astrocytes (glial fibrillary acidic protein (GFAP)), neurons (neuronal nuclei (NeuN)), oligodendrocytes (adenomatous polyposis coli (APC)), axons (neurofilament 200 (NF200)), microglia/macrophages (OX-42) and glial progenitors (NG2). The sections were processed following the same protocol detailed in a previous report [43]. Fz5 was visualised by using the same primary antibody detailed above for single immunohistochemistry at a dilution of 1∶50 and the corresponding Dylight594-linked anti-rabbit secondary antibody (1∶500) (Abcam, ab96897). The sections were then incubated with the following primary antibodies produced in mouse: anti-GFAP (1∶1000) (Sigma, G3893), anti-NeuN (1∶250) (Millipore, MAB377), anti-APC (1∶100) (Calbiochem, OP80), anti-NF200 (1∶2000) (Sigma, N0142), anti-OX42 (1∶500) (Abcam, ab58457) and anti-NG2 (1∶300) (Zymed, 37–2700). Finally, the corresponding Dylight488-linked anti-mouse secondary antibody was used (1∶500) (Abcam, ab96879).

The sections processed for single immunohistochemistry were examined on a BX61 Motorized Research Microscope (Olympus), while the double-labelled sections were analysed using both the BX61 Motorized Research Microscope (Olympus) and a Leica TCS SP5 confocal microscope (Leica Microsystems). For single immunohistochemistry experiments, the sections processed without the primary antibody were used as controls. For double immunohistochemistry, in order to confirm a lack of cross-reactivity, the sections were processed without the second primary antibody and used as controls. No non-specific staining was observed.

### Statistical analysis

All values are expressed as the mean ± SEM. Statistical comparisons of Fz mRNAs expression were performed using a one-way ANOVA followed by Tukey's post-hoc test to determine the individual differences between the means. In all cases p<0.05 was considered statistically significant. All statistical analyses were performed using GraphPad Prism (version 4.0).

## Results

### Temporal mRNA expression pattern of Fz receptors in the NL spinal cord and after contusive SCI

**Figure 1:**
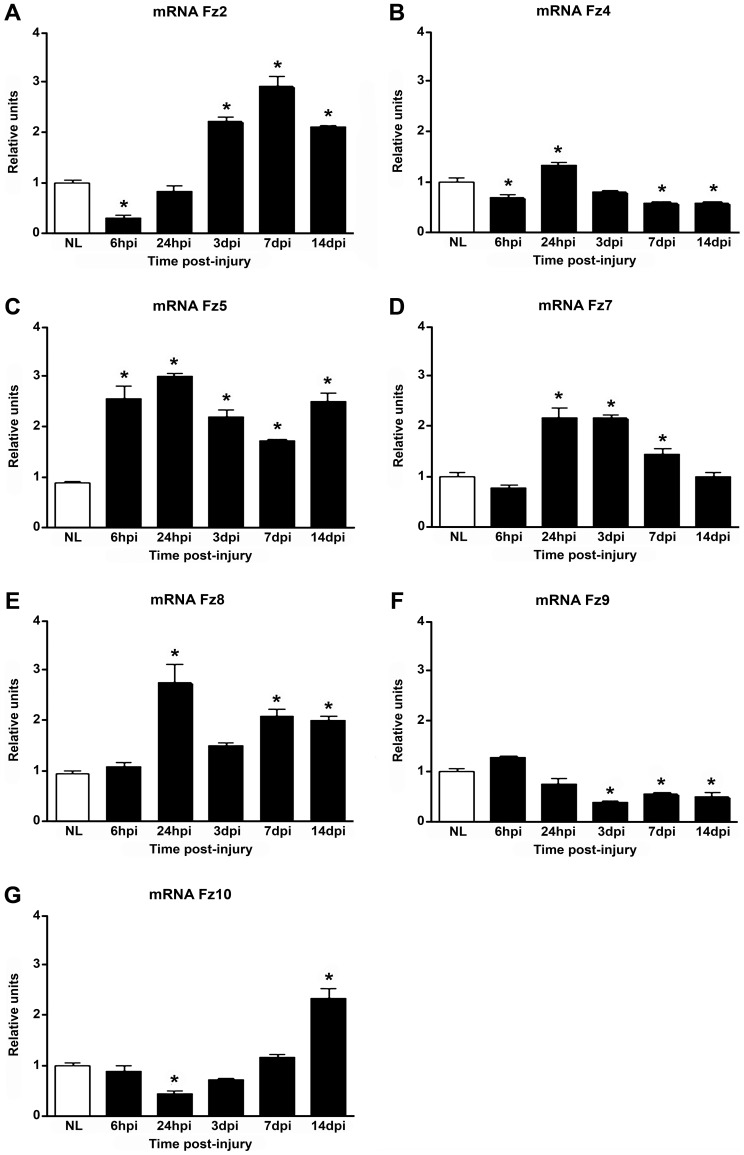
Temporal mRNA expression pattern of Frizzled receptors in the non-lesioned spinal cord and after SCI. The temporal mRNA expression pattern of *Fz2* (1A), *Fz4* (1B), *Fz5* (1C), *Fz7* (1D), *Fz8* (1E), *Fz9* (1F) and *Fz10* (1G) was quantified using quantitative RT-PCR using specific primers in the non-lesioned (NL) spinal cord and at different time points after spinal cord injury. Differences were calculated by setting the expression values of the NL samples at 1 and normalising against ribosomal 18S rRNA. In all cases, the data are presented as the mean ± SEM; * p<0.05 versus NL. hpi, hours post-injury; dpi, days post-injury; Fz, Frizzled.

Using RT-qPCR, we first evaluated the temporal mRNA expression pattern of the Fz receptors [8]. As shown in Figure 1, the mRNAs encoding most Fz receptors (*Fz2*, -*4*, -*5*, -*7*, -*8*, -*9* and -*10*) were detected in the NL spinal cords and after contusive SCI. Only the *Fz1*, -*3* and -*6* mRNAs showed undetectable levels of expression (Ct over 35) both under physiological conditions and after SCI. Moreover, the mRNA expression pattern of the detectable Fz receptors changed during the temporal progression of the contusive SCI.

Specifically, *Fz2* mRNA was significantly down-regulated at 6 hpi, whereas at 24 hpi it returned to the basal level detected in the NL spinal cords (Figure 1A). At 3 dpi, there was a significant increase in *Fz2* mRNA, with a peak of expression at 7 dpi (∼2.8-fold change) that remained significantly up-regulated at 14 dpi compared with NL control animals (Figure 1A). *Fz4* mRNA was down-regulated at 6 hpi, followed by a significant increase at 24 hpi (Figure 1B). However, at 3 dpi *Fz4* mRNA expression returned to the basal level detected in the NL control animals, followed by a significant decrease in its expression from 7 to 14 dpi (Figure 1B). *Fz5* mRNA was significantly increased at all of the analysed post-injury time points compared to the NL control animals (Figure 1C). However, its mRNA expression displayed a biphasic temporal pattern: it peaked at 6 and 24 hpi (∼2.6- and 3-fold changes, respectively) and finally at 14 dpi (∼2.5-fold change) (Figure 1C). As shown in Figure 1D, there was a significant up-regulation of *Fz7* mRNA at 24 hpi and at 3 dpi (∼2.1-fold changes). At 7 dpi *Fz7* started to decrease, and it returned to the basal level detected in the NL spinal cords at 14 dpi (Figure 1D). Similar to *Fz5*, *Fz8* mRNA exhibited a biphasic temporal expression pattern with an early (∼2.7-fold change at 24 hpi) and a late peak of expression (∼2.1-fold changes at 7 and 14 dpi) (Figure 1E). However, no changes in *Fz8* mRNA were observed at 6 hpi or 3 dpi compared with the NL control animals (Figure 1E). No changes in *Fz9* mRNA expression were observed at 6 or 24 hpi (Figure 1F), but it was significantly down-regulated from 3 to 14 dpi (Figure 1F). Finally, compared with NL controls, no variation in *Fz10* mRNA expression was detected at 6 hpi, while a significant decrease was observed at 24 hpi (Figure 1G). Thereafter, *Fz10* mRNA expression returned to the basal level observed in the NL control animals at 3 and 7 dpi but significantly increased at 14 dpi (∼2.3-fold change) (Figure 1G).

### Spatio-temporal protein expression pattern of Fz receptors in the NL spinal cord and after contusive SCI

We next assessed whether the changes in the Fz mRNAs detected above were reflected at the protein level by using single immunohistochemistry. As detailed below, all of these Fz receptors (Fz2, 4, 5, 7, 8, 9 and 10) were expressed under physiological conditions and after SCI. Moreover, their spatio-temporal expression patterns changed almost completely in the sections corresponding to the impact site, whereas no variations were observed in regions that were distant from the injury epicentre. For this reason, the detailed description of the histological data refers to the sections corresponding to the lesion epicentre.

#### Fz2

**Figure 2:**
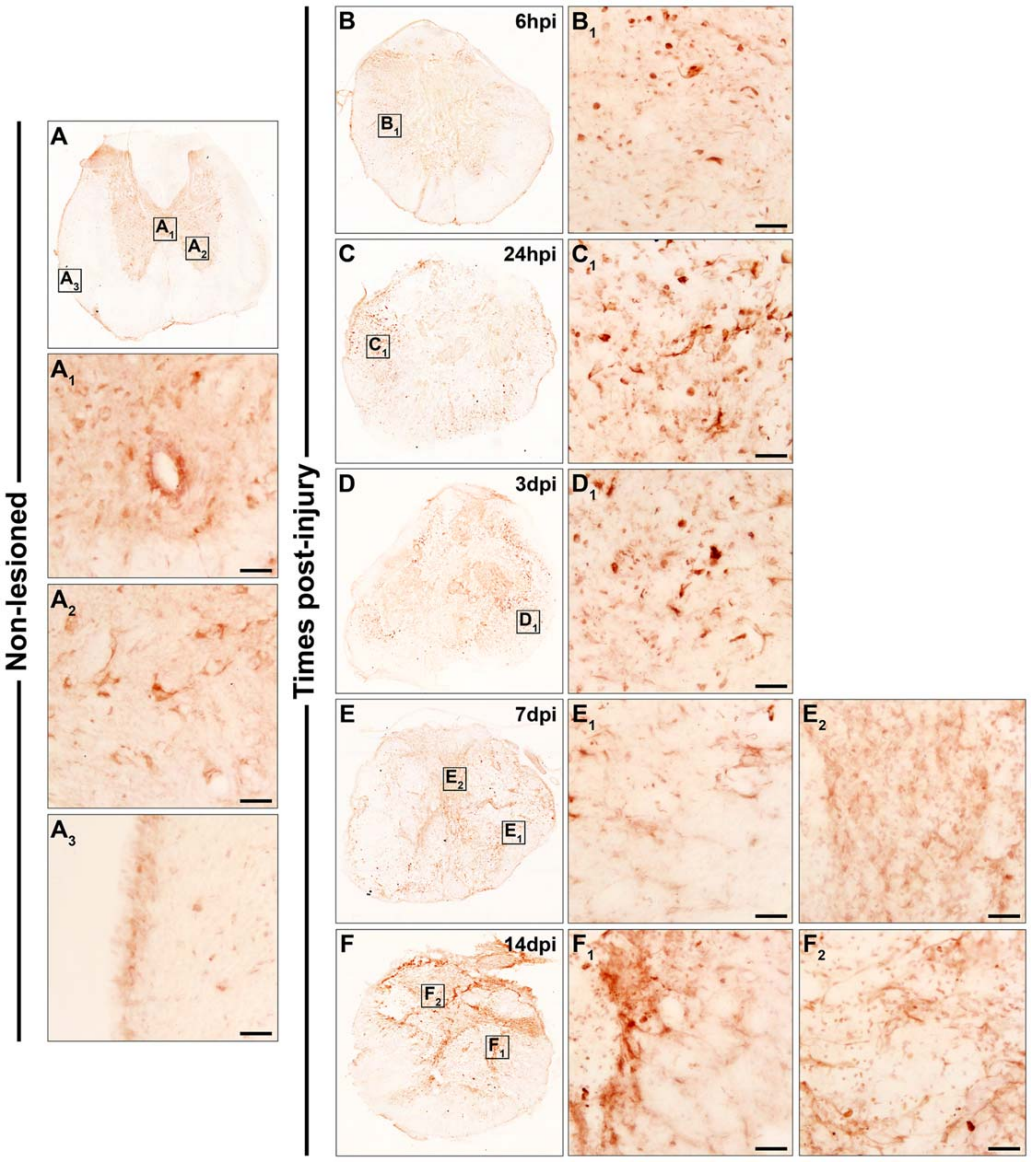
Spatio-temporal protein expression pattern of Frizzled 2 in the non-lesioned spinal cord and after SCI. This figure shows representative images obtained from the microscopic evaluation of sections processed by single immunohistochemistry to visualise Fz2. The analysis was performed in non-lesioned spinal cords (2A, A_1_, A_2_ and A_3_) and lesioned spinal cords at 6 hpi (2B and B_1_) and 24 hpi (2C and C_1_) and at 3 dpi (2D and D_1_), 7 dpi (2E, E_1_ and E_2_) and 14 dpi (2F, F_1_ and F_2_). The squares in the images showing the entire spinal cord sections correspond to the areas of higher magnification. Scale bars = 50 µm. hpi, hours post-injury; dpi, days post-injury; Fz, Frizzled.

As shown in Figure 2A, in the NL spinal cords, Fz2 immunolabelling was prominently observed in central canal cells (Figure 2A_1_) and in grey matter cells displaying a neuronal-like profile (Figure 2A_2_). In the white matter, small and poorly ramified Fz2^+^ cells were detected (Figure 2A_3_). At 6 hpi (Figure 2B and B_1_), 24 hpi (Figure 2C and C_1_), and 3 dpi (Figure 2D and D_1_), Fz2^+^ cells with either a rounded or elongated morphology were observed in the periphery of the sections, although their presence was more evident at 24 hpi and 3 dpi. At 7 (Figure 2E, E_1_ and E_2_) and 14 dpi (Figure 2F, F_1_ and F_2_), Fz2^+^ cells mainly displaying either a highly ramified morphology or a rounded profile were detected both in the periphery (mainly in contact with the lesioned tissue) (Figure 2E, E_1_, F and F_1_) and in the centre of the sections (Figure 2E, E_2_, F and F_2_).

#### Fz4

**Figure 3:**
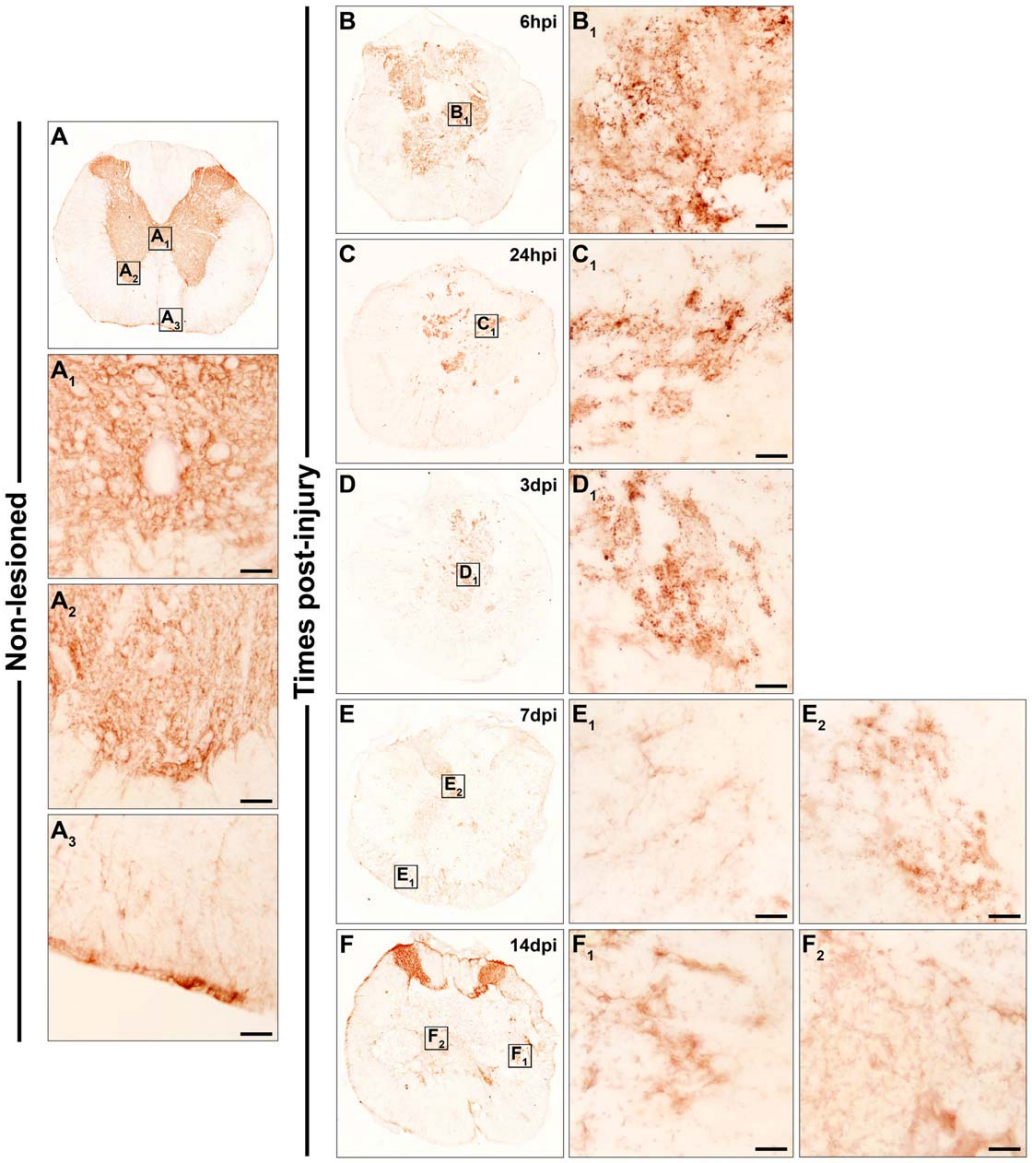
Spatio-temporal protein expression pattern of Frizzled 4 in the non-lesioned spinal cord and after SCI. This figure shows representative images obtained from the microscopic evaluation of sections processed by single immunohistochemistry to visualise Fz4. The analysis was performed in non-lesioned spinal cords (3A, A_1_, A_2_ and A_3_) and lesioned spinal cords at 6 hpi (3B and B_1_) and 24 hpi (3C and C_1_) and at 3 dpi (3D and D_1_), 7 dpi (3E, E_1_ and E_2_) and 14 dpi (3F, F_1_ and F_2_). The squares in the images showing the entire spinal cord sections correspond to the areas of higher magnification. Scale bars = 50 µm. hpi, hours post-injury; dpi, days post-injury; Fz, Frizzled.

In the NL spinal cords (Figure 3A), Fz4 immunolabelling was observed in highly ramified cells in both the grey (Figure 3A_2_) and white matter (Figure 3A_3_), but not in central canal cells (Figure 3A_1_). At 6 hpi (Figure 3B and B_1_), 24 hpi (Figure 3C and C_1_), and 3 dpi (Figure 3D and D_1_), a fine and punctate Fz4 immunolabelling was detected in the centre of the sections, whereas ramified Fz4^+^ cells were still present in the periphery. At 7 dpi, the presence of ramified Fz4^+^ cells, mainly in contact with the damaged tissue, was more evident (Figure 3E and E_1_). In the centre of the sections, the fine punctate Fz4 immunolabelling was still observed in small areas (Figure 3E and E_2_). At 14 dpi, the Fz4 expression pattern was similar to 7 dpi (Figure 3F, F_1_ and F_2_), although in the centre of the sections Fz4 staining was predominantly observed in cells displaying a ramified profile (Figure 3F and F_2_).

#### Fz5

**Figure 4:**
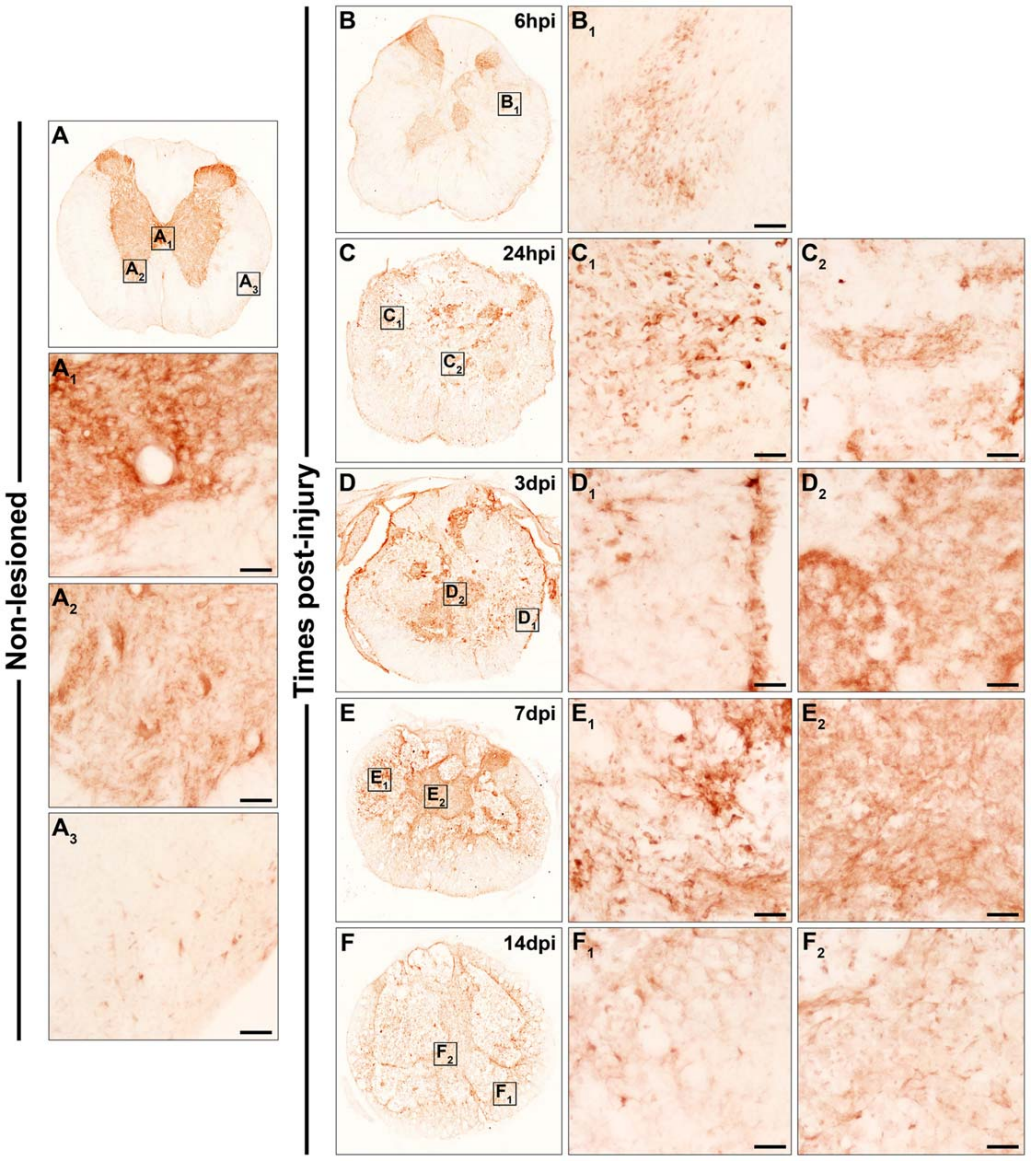
Spatio-temporal protein expression pattern of Frizzled 5 in the non-lesioned spinal cord and after SCI. This figure shows representative images obtained from the microscopic evaluation of sections processed by single immunohistochemistry to visualise Fz5. The analysis was performed in non-lesioned spinal cords (4A, A_1_, A_2_ and A_3_) and lesioned spinal cords at 6 hpi (4B and B_1_) and 24 hpi (4C, C_1_ and C_2_) and at 3 dpi (4D, D_1_ and D_2_), 7 dpi (4E, E_1_ and E_2_) and 14 dpi (4F, F_1_ and F_2_). The squares in the images showing the entire spinal cord sections correspond to the areas of higher magnification. Scale bars = 50 µm. hpi, hours post-injury; dpi, days post-injury; Fz, Frizzled.

As shown in Figure 4A, Fz5 immunolabelling in the NL spinal cord was observed in central canal cells (Figure 4A_1_) and in grey matter cells displaying neuronal-like or small ramified profiles (Figure 4A_2_). In the white matter, small ramified Fz5^+^ cells were also detected (Figure 4A_3_). At 6 hpi, few clusters of small rounded or elongated cells expressing Fz5 were observed (Figure 4B and B_1_). At 24 hpi in the periphery of the sections (Figure 4C and C_1_), Fz5 was still expressed in cells with a rounded morphology and also in cells showing an elongated profile, whereas in the centre of these sections (Figure 4C and C_2_) several ramified Fz5^+^ cells were detected together with a fine and punctuated Fz5 immunolabelling. At 3 (Figure 4D, D_1_ and D_2_), 7 (Figure 4E, E_1_ and E_2_) and 14 dpi (Figure 4F, F_1_ and F_2_), Fz5 immunolabelling was observed in ramified cells in the peripheral areas of the sections (mainly in contact with the injured tissue) (Figure 4D_1_, E_1_ and F_1_) and in either rounded or ramified cells in the centre of these same sections (Figure 4D_2_, E_2_ and F_2_). However, at 3 dpi the fine and punctuate Fz5 immunolabelling was still observed in the centre of the sections (Figure 4D and D_2_).

#### Fz7

**Figure 5:**
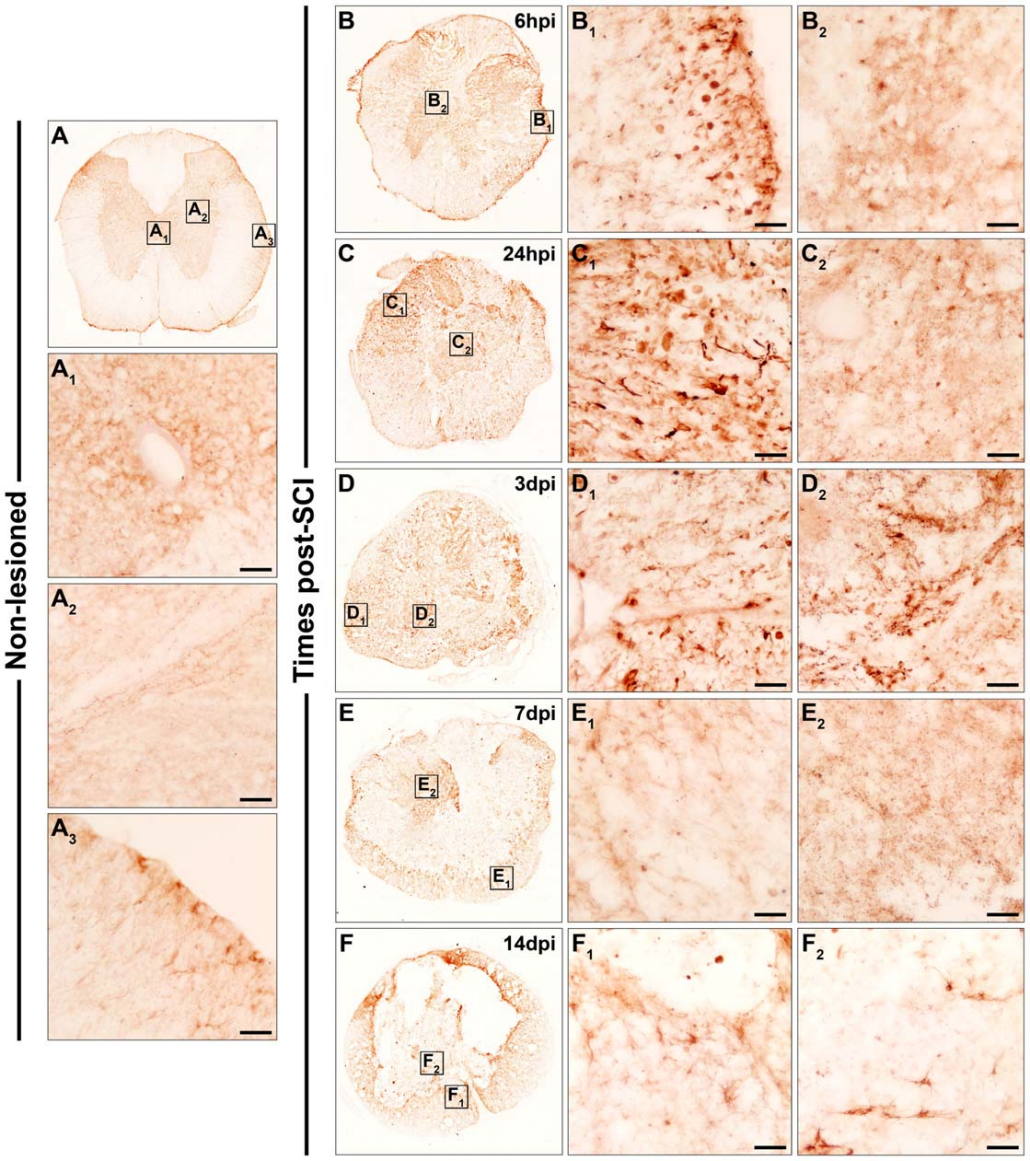
Spatio-temporal protein expression pattern of Frizzled 7 in the non-lesioned spinal cord and after SCI. This figure shows representative images obtained from the microscopic evaluation of sections processed by single immunohistochemistry to visualise Fz7. The analysis was performed in non-lesioned spinal cords (5A, A_1_, A_2_ and A_3_) and lesioned spinal cords at 6 hpi (5B, B_1_ and B_2_) and 24 hpi (5C, C_1_ and C_2_) and at 3 dpi (5D, D_1_ and D_2_), 7 dpi (5E, E_1_ and E_2_) and 14 dpi (5F, F_1_ and F_2_). The squares in the images showing the entire spinal cord sections correspond to the areas of higher magnification. Scale bars = 50 µm. hpi, hours post-injury; dpi, days post-injury; Fz, Frizzled.

In the NL spinal cords (Figure 5A), no Fz7^+^ central canal cells were detected (Figure 5A_1_), while Fz7 was observed in the grey matter in faintly stained cells that showed a ramified morphology. Fz7 was also expressed in fine and extremely elongated structures with small engrossments that not only crossed the intermediate grey matter (Figure 5A_2_) but also appeared in the ventral and dorsal horns. In the white matter, highly ramified Fz7^+^ cellular profiles were present (Figure 5A_3_). At 6 hpi (Figure 5B, B_1_ and B_2_), 24 hpi (Figure 5C, C_1_ and C_2_), and 3 dpi (Figure 5D, D_1_ and D_2_), ramified, rounded or elongated Fz7^+^ cells were observed in the periphery of the sections (Figure 5B_1_, C_1_ and D_1_), whereas in the centre (Figure 5B_2_, C_2_ and D_2_) Fz7^+^ cells displaying a ramified or, to a lesser extent, rounded morphology were detected, together with a fine punctate Fz7 immunolabelling. At 7 dpi, the presence of ramified cells expressing Fz7 was more evident in the periphery of the sections (mainly in contact with the injured tissue) (Figure 5E and E_1_), while in the centre (Figure 5E and E_2_) small areas were occupied by ramified Fz7^+^ cells and a fine punctate Fz7 staining. At 14 dpi (Figure 5F, F_1_ and F_2_), mainly ramified Fz7^+^ cells were observed, prominently in the peripheral areas of these sections (Figure 5F_1_).

#### Fz8

**Figure 6:**
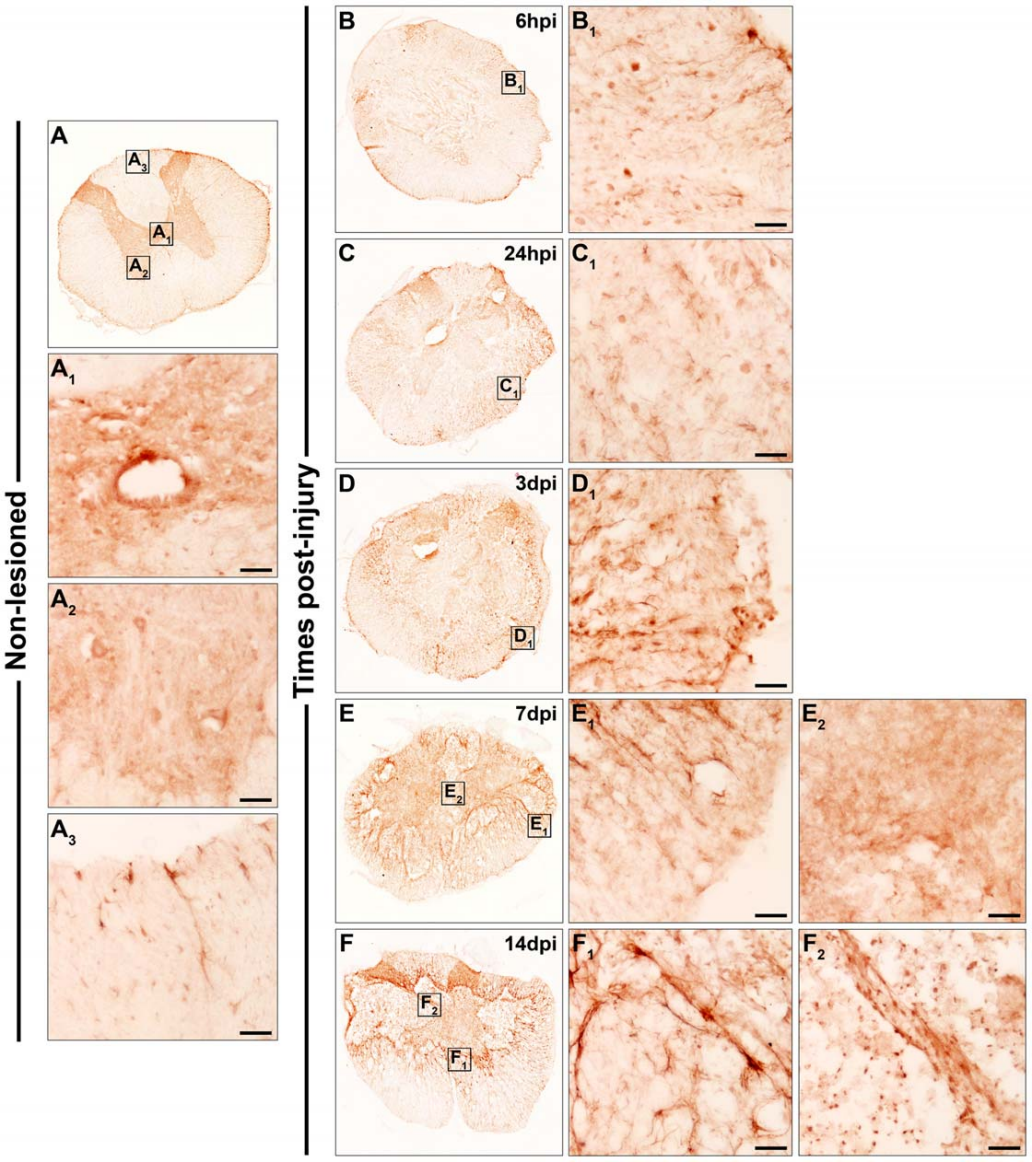
Spatio-temporal protein expression pattern of Frizzled 8 in the non-lesioned spinal cord and after SCI. This figure shows representative images obtained from the microscopic evaluation of sections processed by single immunohistochemistry to visualise Fz8. The analysis was performed in non-lesioned spinal cords (6A, A_1_, A_2_ and A_3_) and lesioned spinal cords at 6 hpi (6B and B_1_) and 24 hpi (6C and C_1_) and at 3 dpi (6D and D_1_), 7 dpi (6E, E_1_ and E_2_) and 14 dpi (6F, F_1_ and F_2_). The squares in the images showing the entire spinal cord sections correspond to the areas of higher magnification. Scale bars = 50 µm. hpi, hours post-injury; dpi, days post-injury; Fz, Frizzled.

In the NL spinal cords (Figure 6A), Fz8 immunolabelling was observed in the grey matter, in cells displaying a neuronal-like morphology (Figure 6A_2_), in small rounded cells (Figure 6A_2_) and in central canal cells (Figure 6A_1_). In the white matter, Fz8 was expressed in cells displaying a highly ramified profile (Figure 6A_3_). At 6 hpi (Figure 6B and B_1_), 24 hpi (Figure 6C and C_1_), and 3 dpi (Figure 6D and D_1_), Fz8 expression was mainly restricted to the periphery of the sections, where Fz8^+^ cells with either a rounded or ramified morphology were observed. At 7 (Figure 6D, D_1_ and D_2_) and 14 dpi (Figure 6E, E_1_ and E_2_), Fz8^+^ cells were located either in the periphery (mainly in contact with the injured tissue) (Figure 6D_1_ and E_1_) or in the centre of the sections (Figure 6D_2_ and E_2_) and mainly exhibited a ramified morphology.

#### Fz9

**Figure 7:**
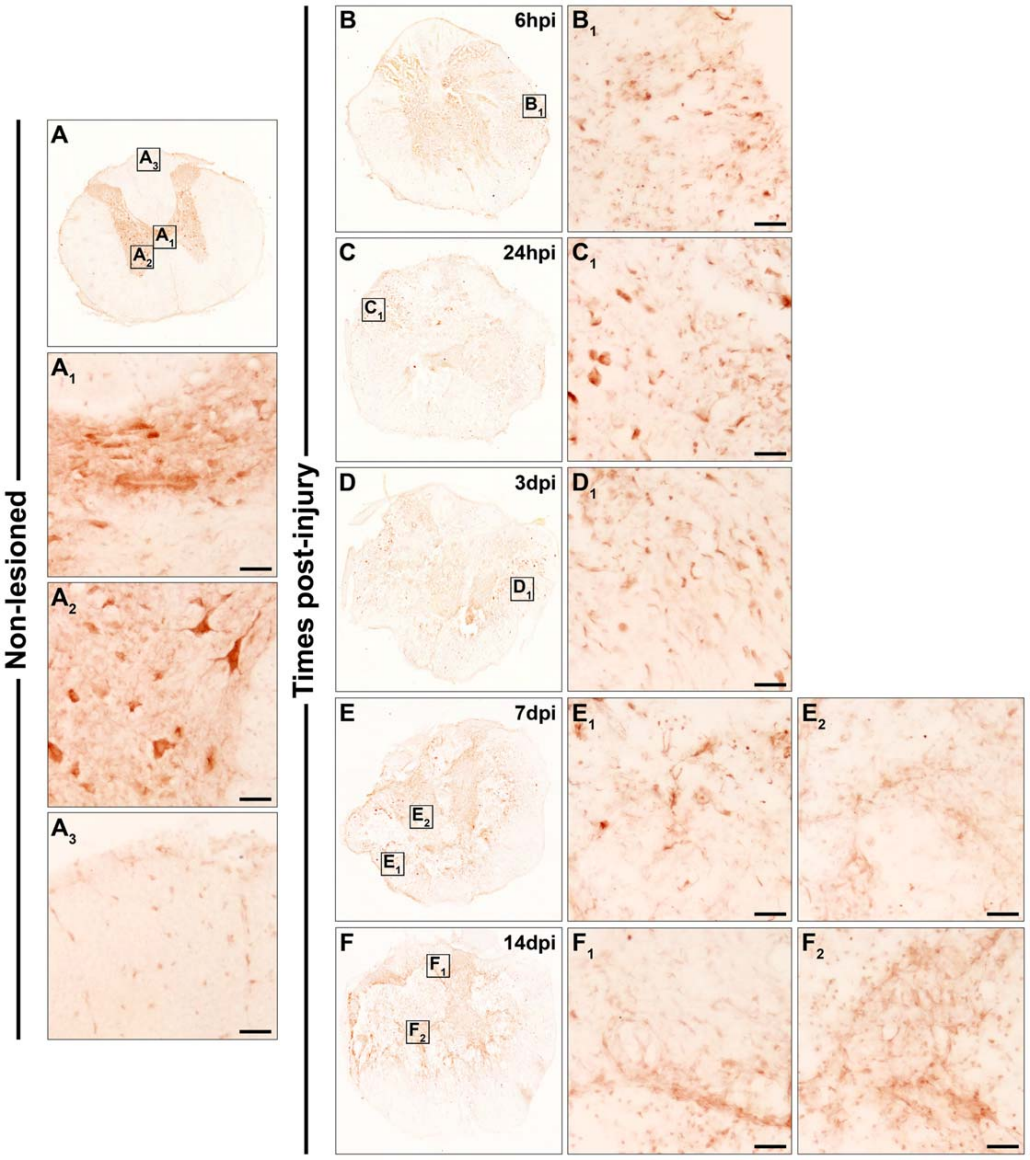
Spatio-temporal protein expression pattern of Frizzled 9 in the non-lesioned spinal cord and after SCI. This figure shows representative images obtained from the microscopic evaluation of sections processed by single immunohistochemistry to visualise Fz9. The analysis was performed in non-lesioned spinal cords (7A, A_1_, A_2_ and A_3_) and lesioned spinal cords at 6 hpi (7B and B_1_) and 24 hpi (7C and C_1_) and at 3 dpi (7D and D_1_), 7 dpi (7E, E_1_ and E_2_) and 14 dpi (7F, F_1_ and F_2_). The squares in the images showing the entire spinal cord sections correspond to the areas of higher magnification. Scale bars = 50 µm. hpi, hours post-injury; dpi, days post-injury; Fz, Frizzled.

As shown in Figure 7A, Fz9 immunolabelling in the NL spinal cords was detected in cells belonging to the central canal (Figure 7A_1_) and in grey matter cells exhibiting a neuronal-like morphology (Figure 7A_2_). Several small and poorly ramified cells expressed Fz9 in the white matter (Figure 7A_3_). At 6 hpi (Figure 7B and B_1_), 24 hpi (Figure 7C and C_1_), and 3 dpi (Figure 7D and D_1_), Fz9^+^ cells, which showed either a rounded or elongated morphology, were located mainly in the periphery of the sections. At 7 dpi (Figure 7E, E_1_ and E_2_) and 14 dpi (Figure 7F, F_1_ and F_2_), Fz9 expressing cells displaying either a ramified or, to a lesser extent, amoeboid profile were located both in the periphery (mainly in contact with the injured tissue) (Figure 7E_1_ and F_1_) and in the centre of the sections (Figure 7E_2_ and F_2_).

#### Fz10

**Figure 8:**
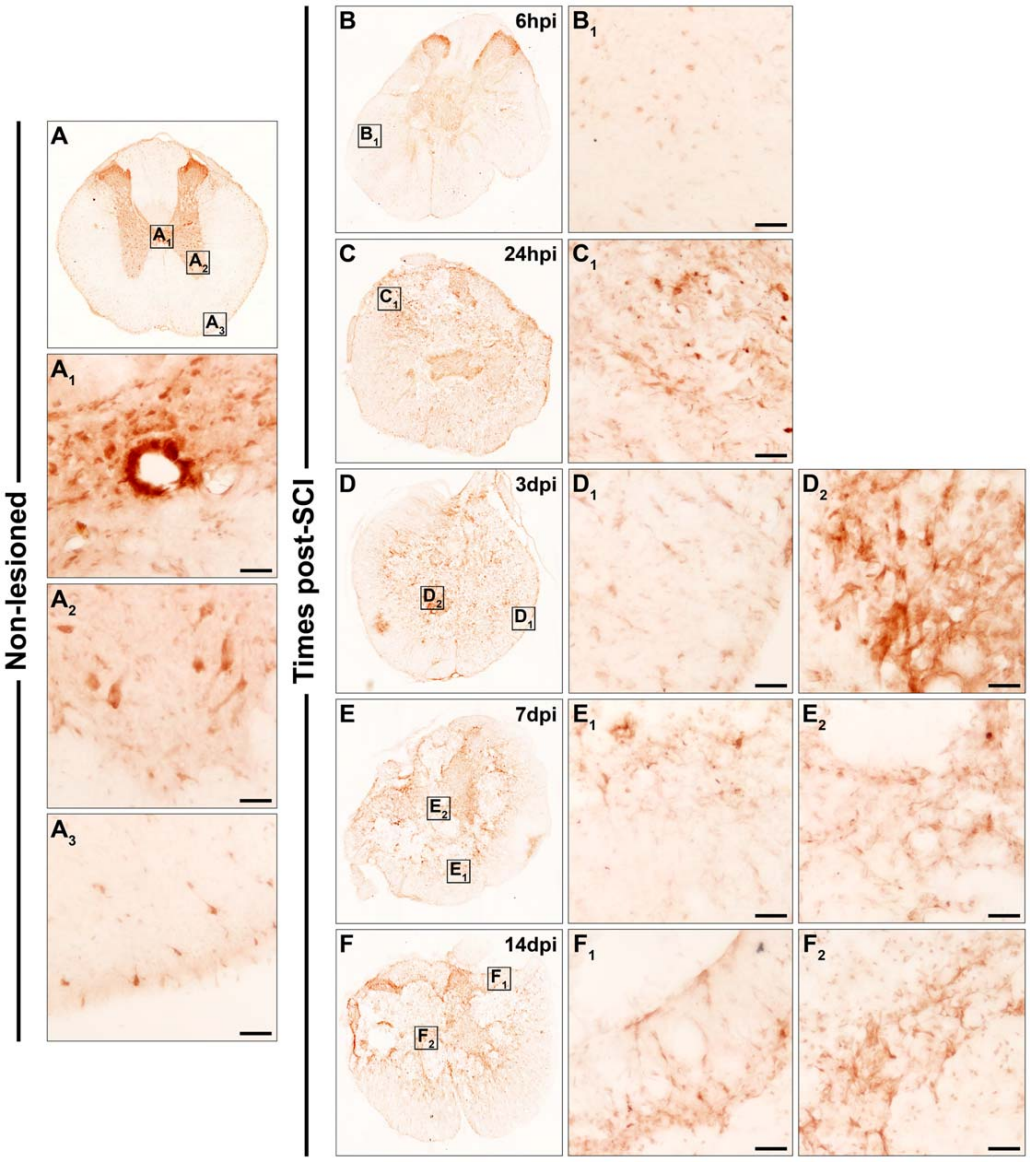
Spatio-temporal protein expression pattern of Frizzled 10 in the non-lesioned spinal cord and after SCI. This figure shows representative images obtained from the microscopic evaluation of sections processed by single immunohistochemistry to visualise Fz10. The analysis was performed in non-lesioned spinal cords (8A, A_1_, A_2_ and A_3_) and lesioned spinal cords at 6 hpi (8B and B_1_) and 24 hpi (8C and C_1_) and at 3 dpi (8D, D_1_ and D_2_), 7 dpi (8E, E_1_ and E_2_) and 14 dpi (8F, F_1_ and F_2_). The squares in the images showing the entire spinal cord sections correspond to the areas of higher magnification. Scale bars = 50 µm. hpi, hours post-injury; dpi, days post-injury; Fz, Frizzled.

Fz10 immunolabelling in the NL spinal cords (Figure 8A) was detected in central canal cells (Figure 8A_1_). Moreover, Fz10 was observed in the grey matter, mainly in cells displaying a neuronal-like morphology (Figure 8A_1_) but also in small and poorly ramified cells (Figure 8A_2_), and in the white matter in small ramified cells (Figure 8A_3_). At 6 hpi (Figure 8B and B_1_) and 24 hpi (Figure 8C and C_1_), Fz10^+^ cells were mainly observed in the periphery of the sections in either rounded or elongated cellular profiles. At 3 dpi (Figure 8D, D_1_ and D_2_), 7 dpi (Figure 8E, E_1_ and E_2_) and 14 dpi (Figure 8F, F_1_ and F_2_), Fz10 was expressed mainly in ramified cells, both in the periphery (mainly in contact with the injured tissue) (Figure 8D_1_, E_1_ and F_1_) and in the centre of the sections (Figure 8D_2_, E_2_ and F_2_).

The results obtained from the evaluation of the spatio-temporal expression patterns of the different Fz receptors by single immunohistochemistry both in the non-lesioned spinal cords and at those analysed times post-SCI are summarized in Table 1.

**Table 1:** Summary of the results obtained from the evaluation of the spatio-temporal expression pattern of Frizzled receptors by single immunohistochemistry.

		TISSUE SECTIONS												
		Grey matter				White matter	Periphery				Centre			
		Neuronal-like	Ramified	Rounded	Elongated	Ramified	Ramified	Rounded	Elongated	Punctated	Ramified	Rounded	Elongated	Punctated
	NL	+				+								
	6 hpi							+	+					
Fz2	24 hpi							+	+					
	3 dpi							+	+					
	7 dpi						+	+			+	+		
	14 dpi						+	+			+	+		
	NL		+			+								
	6 hpi						+							+
Fz4	24 hpi						+							+
	3 dpi						+							+
	7 dpi						+							+
	14 dpi						+				+			+
	NL	+	+			+								
	6 hpi							+	+					
Fz5	24 hpi							+	+		+			+
	3 dpi						+				+	+		+
	7 dpi						+				+	+		
	14 dpi						+				+	+		
	NL		+		+	+								
	6 hpi						+	+	+		+	+	+	+
Fz7	24 hpi						+	+	+		+	+	+	+
	3 dpi						+	+	+		+	+	+	+
	7 dpi						+				+			+
	14 dpi						+				+			
	NL	+		+		+								
	6 hpi						+	+						
Fz8	24 hpi						+	+						
	3 dpi						+	+						
	7 dpi						+				+			
	14 dpi						+				+			
	NL	+				+								
	6 hpi							+	+					
Fz9	24 hpi							+	+					
	3 dpi							+	+					
	7 dpi						+	+			+	+		
	14 dpi						+	+			+	+		
	NL	+	+			+								
	6 hpi							+	+					
Fz10	24 hpi							+	+					
	3 dpi						+				+			
	7 dpi						+				+			
	14 dpi						+				+			

This table shows a summary of the different profiles (neuronal-like, ramified, rounded, elongated and punctated) found in the different areas evaluated for each Frizzled (Fz) receptor, both in the non-lesioned (NL) spinal cords and at the different times post-SCI. In accordance with the Results section, the tissue sections corresponding to the NL spinal cords are subdivided into grey and white matter, while the tissue sections corresponding to the lesion epicentre in lesioned spinal cords are subdivided in periphery and centre. hpi, hours post-injury; dpi, days post-injury. +indicates the presence of Fz staining.

### Cellular protein expression pattern of Fz5 receptor in the NL spinal cord and after contusive SCI

To evaluate which specific cell types expressed Fz receptors and whether the changes observed in their spatio-temporal expression patterns implied variations in their cellular expression patterns, we have analysed the cellular Fz5 expression both in the NL spinal cords and after SCI, since this receptor seems to be the most up-regulated member of the Fz family of proteins at all evaluated times post-SCI.

**Figure 9:**
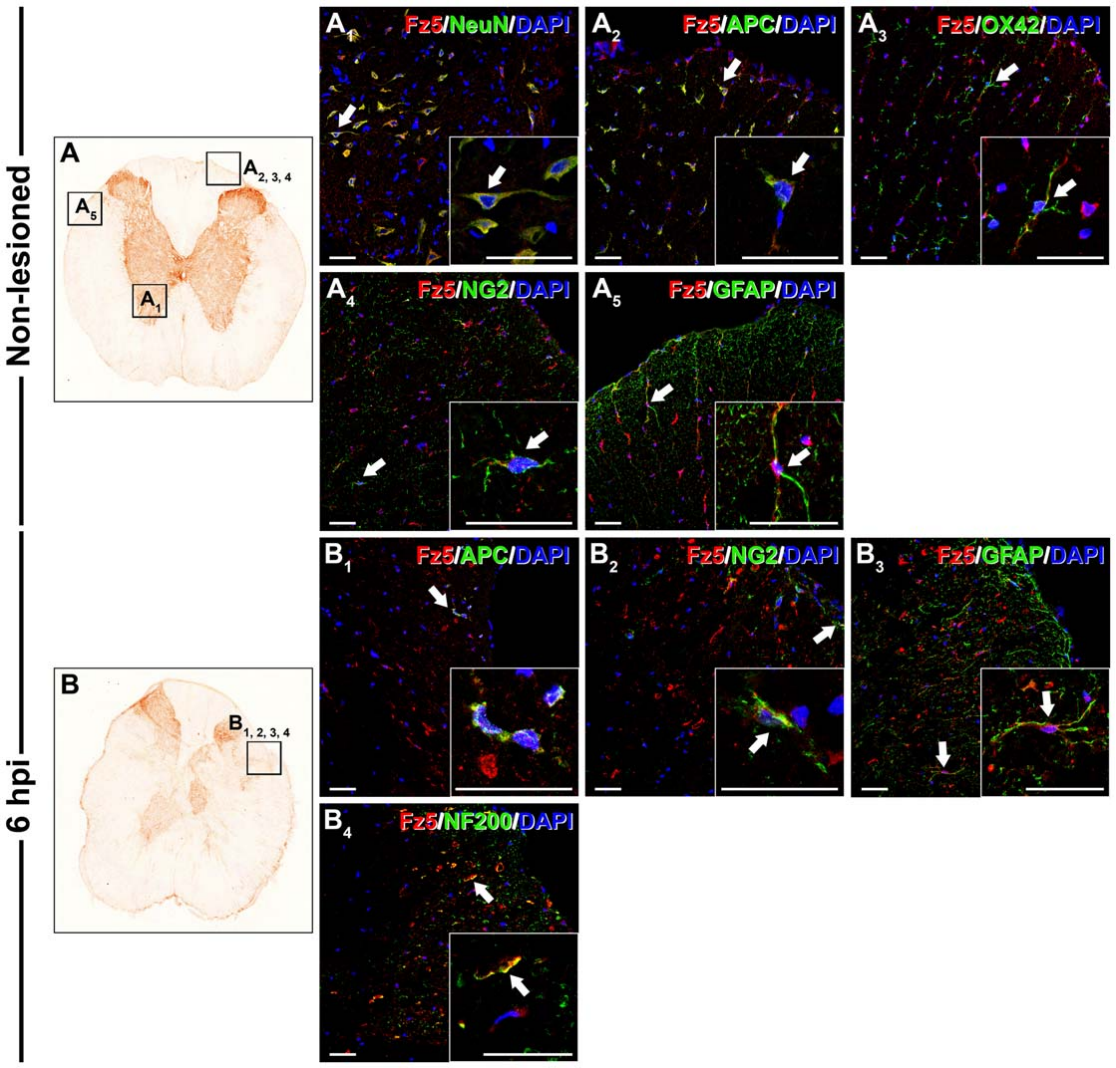
Cellular protein expression pattern of Frizzled 5 in non-lesioned spinal cords and at 6 hpi. This figure shows representative images obtained from the microscopic evaluation of sections processed by double immunohistochemistry to visualise Frizzled (Fz) 5 in astrocytes (glial fibrillary acidic protein (GFAP)), neurons (neuronal nuclei (NeuN)), oligodendrocytes (adenomatous polyposis coli (APC)), axons (neurofilament 200 (NF200)), microglia/macrophages (OX-42) and glial progenitors (NG2) in the non-lesioned spinal cords (9A, A_1_, A_2_, A_3_, A_4_ and A_5_) and at 6 hours post-injury (hpi) (9B, B_1_, B_2_, B_3_ and B_4_). The squares in the images showing the entire spinal cord sections (9A and B) correspond to the areas of higher magnification. Scale bars = 40 µm.

In the NL spinal cords (Figure 9A) Fz5 expression was detected in neurons located in all spinal cord laminae (Figure 9A_1_), oligodendrocytes (Figure 9A_2_), quiescent microglial cells (Figure 9A_3_), NG2^+^ glial precursors (Figure 9A_4_) and astrocytes (Figure 9A_5_), but not in axonal projections.

**Figure 10:**
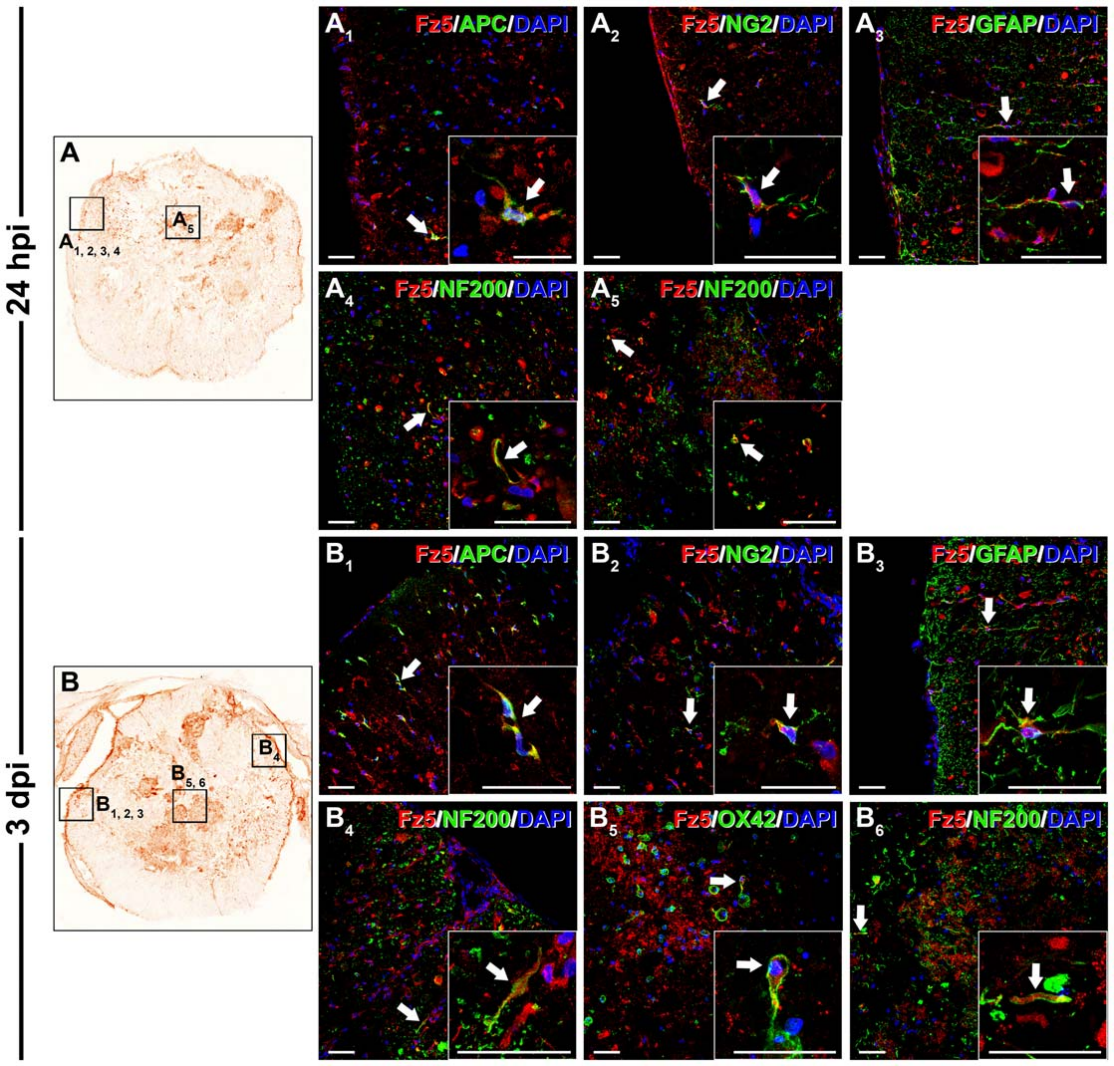
Cellular protein expression pattern of Frizzled 5 at 24 hpi and 3 dpi. This figure shows representative images obtained from the microscopic evaluation of sections processed by double immunohistochemistry to visualise Frizzled (Fz) 5 in astrocytes (glial fibrillary acidic protein (GFAP)), neurons (neuronal nuclei (NeuN)), oligodendrocytes (adenomatous polyposis coli (APC)), axons (neurofilament 200 (NF200)), microglia/macrophages (OX-42) and glial progenitors (NG2) at 24 hours post-injury (hpi) (10A, A_1_, A_2_, A_3_, A_4_ and A_5_) and 3 days post-injury (dpi) (10B, B_1_, B_2_, B_3_, B_4_, B_5_ and B_6_). The squares in the images showing the entire spinal cord sections (10A and B) correspond to the areas of higher magnification. Scale bars = 40 µm.

**Figure 11:**
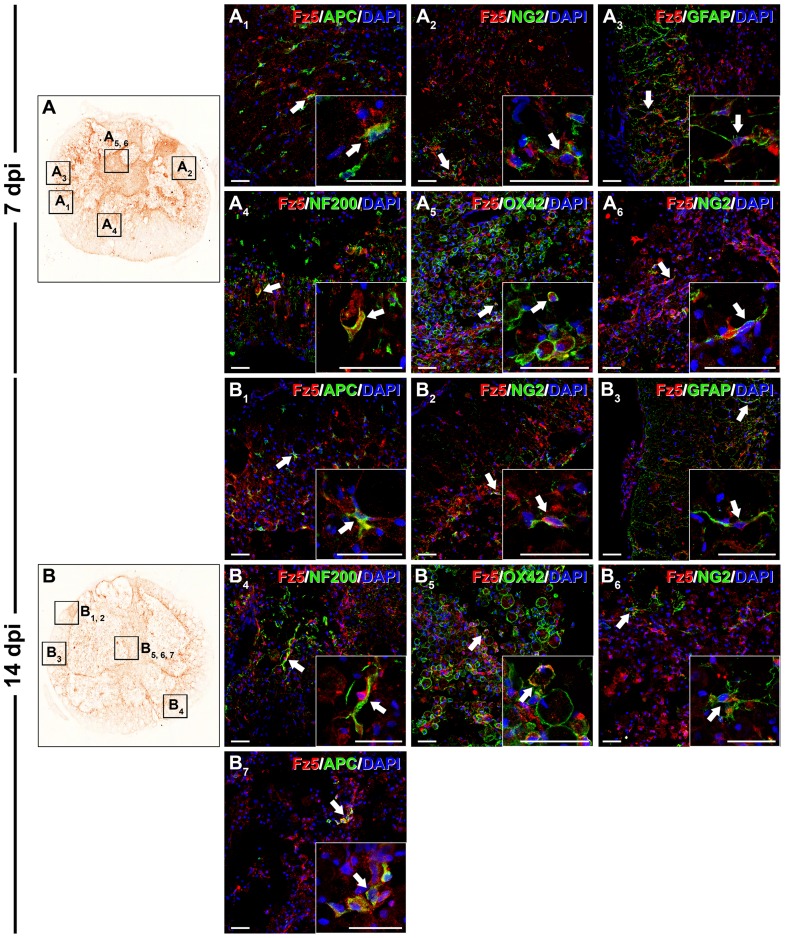
Cellular protein expression pattern of Frizzled 5 at 7 and 14 dpi. This figure shows representative images obtained from the microscopic evaluation of sections processed by double immunohistochemistry to visualise Frizzled (Fz) 5 in astrocytes (glial fibrillary acidic protein (GFAP)), neurons (neuronal nuclei (NeuN)), oligodendrocytes (adenomatous polyposis coli (APC)), axons (neurofilament 200 (NF200)), microglia/macrophages (OX-42) and glial progenitors (NG2) at 7 (11A, A_1_, A_2_, A_3_, A_4_, A_5_ and A_6_) and 14 days post-injury (dpi) (11B, B_1_, B_2_, B_3_, B_4_, B_5_, B_6_ and B_7_). The squares in the images showing the entire spinal cord sections (11A and B) correspond to the areas of higher magnification. Scale bars = 40 µm.

At 6 hpi (Figure 9B), Fz5 was still expressed in oligodendrocytes (Figure 9B_1_), NG2^+^ glial precursors (Figure 9B_2_) and astroglial cells (Figure 9B_3_), while some axons began to express Fz5 (Figure 9B_4_). No Fz5 expression was detected in reactive microglia/macrophages. At 24 hpi (Figure 10A), the cell types expressing Fz5 in the periphery of the sections were identified as oligodendrocytes (Figure 10A_1_), NG2^+^ glial precursors (Figure 10A_2_) and astrocytes (Figure 10A_3_). At this time post-SCI few Fz5^+^ axons were still present, although they were not only located in the periphery (Figure 10A_4_) but also in the centre of the sections (Figure 10A_5_). Similar to those observed at 6 hpi, no Fz5^+^ reactive microglia/macrophages were observed in the damaged areas. At 3 dpi (Figure 10B) and in the periphery of the sections, Fz5 expression was still observed in oligodendroglial cells (Figure 10B_1_), NG2^+^ glial precursors (Figure 10B_2_), astrocytes (Figure 10B_3_) and axonal projections (Figure 10B_4_). In the centre of the sections, Fz5^+^ axons were detected (Figure 10B_6_), while some reactive microglia/macrophages began to express this receptor (Figure 10B_5_). At 7 (Figure 11A) and 14 dpi (Figure 11B) and in the periphery of the sections corresponding to the lesion epicentre, Fz5 was still ^expressed^ in oligodendrocytes (Figure 11A_1_ and 11B_1_), NG2^+^ glial precursors (Figure 11A_2_ and 11B_2_) astrocytes (Figure 11A_3_ and 11B_3_), axons (Figure 11A_4_ and 11B_4_) and reactive microglia/macrophages. In the centre of the sections, Fz5 was expressed in reactive microglia/macrophages (Figure 11A_5_ and B_5_) and NG2^+^ glial precursors (Figure 11A_6_ and B_6_), while at 14 dpi some Fz5^+^ oligodendroglial cells were observed (Figure 11B_7_).

## Discussion

In the present study we demonstrated that mRNAs encoding most of the 10 known Fz receptors (*Fz2*, -*4*, -*5*, -*7*, -*8*, -*9* and -*10*) were expressed in the NL adult rat spinal cord. Moreover, the mRNA expression of Fz receptors was reflected at the protein level, as detected using single immunohistochemistry, and interestingly, the Fz proteins exhibited distinct spatio-temporal expression patterns. Furthermore, our results showed that Fz5 in the NL spinal cords was expressed in different cell types such as neurons, astrocytes, oligodendrocytes, microglial cells and NG2^+^ glial precursors.

Although some conflicting results have been obtained [38], it should be noted that another study has demonstrated the mRNA expression of all Fz receptors in the uninjured adult mouse spinal cord, using *in situ* hybridisation [46]. In that report, all Fz receptors showed a similar spatial mRNA expression pattern, being mainly observed both in the grey matter (in neuronal-like profiles and in central canal cells) and in the white matter (in cells displaying a glial-like morphology) [46]. Although these observations may correlate well with our results regarding the spatial protein expression pattern of Fz2, -8, -9 and -10, there are some intriguing differences. Specifically, we detected no Fz4^+^ or Fz7^+^ neuronal-like or central canal cells, but we did observe many small and ramified Fz5^+^ cells in the grey matter that were identified as glial cells. These discrepancies may reflect between-species differences and/or the existence of post-transcriptional regulatory mechanisms in specific spinal cord anatomical areas and cell types. Therefore, further studies are needed to explain the mechanisms underlying these differences.

Although the functions of Fz receptors in the adult CNS under physiological conditions are still poorly understood, progress has been made recently regarding the involvement of the Wnt family of proteins in many CNS processes, such as synaptic function [10], dendritic arborisation [10], neurogenesis [10], [47], neuronal viability [29] and blood-brain barrier (BBB) maintenance [48]. Nevertheless, although it is tempting to hypothesise that the expression of the different Fz receptors in central canal cells and in cells displaying both glial or neuronal-like morphology in the uninjured spinal cord may be implicated in these important events, our results do not allow us to conclude which roles these receptors might play in the uninjured spinal cord. However, the fact that almost all known Fz receptors, together with most Wnt proteins [42], are physiologically expressed in the spinal cord with specific expression patterns clearly argues for the involvement of these molecules in normal spinal cord functioning during adulthood, beyond their well-known developmental roles [1], [9]. The specific functions that may be mediated by Fz receptors in the NL spinal cord should be experimentally evaluated in future studies.

An increasing amount of experimental evidence suggests the involvement of Wnt family of proteins in the progression of some neuropathologies, including SCI [36], [38], [40]. In agreement with this, we have previously demonstrated that the expression patterns of almost all Wnt ligands and inhibitors change after contusive SCI [42]. To expound upon these results, we showed here that both the mRNA and protein expression of Fz2, -4, -5, -7, -8, -9 and -10 was maintained after injury and that the spatio-temporal expression patterns were altered in this model. Moreover, our results have demonstrated that, as compared with those observed in NL spinal cords, the cellular expression pattern of Fz5 suffered evident variations after SCI, being observed in oligodendrocytes, reactive astrocytes and microglia/macrophages, NG2^+^ glial precursors and axonal projections.

To date, only a single previous report has attempted to analyse the expression of Fz receptors after SCI [38]. Using *in situ* hybridisation, those authors showed that after spinal cord hemisection in mice, only *Fz1* mRNA expression was induced at 1 dpi and declined at 7 dpi [38]. The discrepancies between their observations and those obtained in the present study may be attributable to the differences between the experimental injury models used and/or the differences in the responses to SCI between rats and mice, as described for other SCI-related biological processes [49].

Although there are no conclusive data regarding Fz functions in the progression of SCI, some indirect evidence from the literature together with the Fz spatio-temporal expression patterns shown here indicate a role for these receptors in the multiple events that characterise this neuropathology.

A major cause of the deficits associated with SCI is the secondary death of neurons and oligodendrocytes and, thus, the disruption of neural circuits that are located in the vicinity of the primary injury core [25]. Along these lines, recent *in vitro* and *in vivo* studies have shown that the Wnt family of proteins is involved in neuron survival after a CNS injury. *In vitro*, Wnt1 and Wnt3a administration reduce cell death induced by serum deprivation in PC12 cells [27] and by β-amyloid toxicity in hippocampal neurons [28], respectively. Moreover, the inhibition of Dkk1, an inhibitor of the Wnt canonical signalling pathway, reduces neuronal cell death in NMDA-treated cortical cultures [19], [21]. *In vivo*, Dkk1 inhibition is neuroprotective in a model of temporal lobe epilepsy induced by kainic acid injection [19], [26] and in different models of cerebral ischaemia [19]–[21]. In addition, neurotoxicity related to Alzheimer disease has been associated with alterations in Wnt signalling pathways [14]. In this regard, our results show that Fz5 was expressed in neurons and oligodendroglial cells, not only in the NL spinal cord but also after injury in the preserved spinal cord parenchyma that surrounded the injured tissue, which are thought to be affected by the secondary cell death processes associated with the progression of SCI. Interestingly, it has been recently reported that Fz5 expression and activity is necessary for neuron survival in the parafascicular nucleus under physiological conditions, strongly suggesting the involvement of this receptor in neural cell survival [29]. Moreover and in the same areas detailed above for Fz5, we observed Fz2, -8, -9 and -10 expression in cells displaying a neuronal-like morphology and Fz2, -4, -7, -8, -9 and -10 expression in white matter cells displaying a glial-like morphology.

Another major hallmark of SCI is the activation of the inflammatory response, which may play both a beneficial and a deleterious role [50], [51]. In this context, microglial reactivity is of utmost importance, as activated microglia/macrophages are involved in cellular debris phagocytosis, antigen presentation, cellular trophic support and the production of a wide range of inflammatory mediators [52], [53]. Notably, Wnt5a binding to Fz5 can augment the production of pro-inflammatory cytokines and chemokines by activated peripheral macrophages [30]–[33]. Moreover, Wnt3a induces a pro-inflammatory profile in cultured microglial-like cells, which express Fz2, -4, -5, -7, -8 and -10 [34], whereas different Wnt ligands trigger the proliferation of these cells [35]. Interestingly, our results showed that Fz5 is expressed in reactive microglia/macrophages that are located in the injured areas from 3 to 14 dpi, corresponding with the peak of microglia/macrophage activation [54] and with an increased production of different Wnt ligands such as Wnt5a after SCI [36], [42]. Moreover, we observed that at different times post-SCI, Fz2, -7, -8, -9 and -10 were expressed in the damaged tissue in cells that may resemble reactive microglia/macrophages, as they display an amoeboid morphology and lack cellular processes.

Astroglial reactivity also plays a critical role in SCI progression [55], [56]. Among other functions, reactive astrocytes migrate towards the damaged area [57], generating a fibrous glial scar, which isolates the injured nervous tissue from the non-injured nervous tissue, provides structural support and aids in the regeneration of the compromised BBB [58]–[60]. Intriguingly, we observed that Fz5 was expressed in astroglial cells located in the damaged areas at all evaluated times post-injury, and that Fz5 expression in astrocytes was more evident at 7 and 14 dpi, when this receptor was mainly expressed in those astroglial cells that were closely related to the unstructured damaged nervous tissue. Moreover, at the last evaluated times post-SCI (7 and 14 dpi), all of the Fz receptors analysed in the present study were expressed in cells in the preserved spinal cord parenchyma (which is closely related to the unstructured tissue of the lesion epicentre) that displayed a small polygonal soma and many long and thick cellular processes. This morphology and location are those of the reactive astrocytes that form the glial scar at these time points after SCI [54].

Despite the previously detailed beneficial nature of the glial scar, its formation represents a great obstacle to axonal regeneration [61] and is therefore one of the most important handicaps in functional recovery after SCI [62]. The involvement of the Wnt family of proteins in the axonal regeneration that takes place after SCI has been consistently supported. For instance, the non-conventional Wnt receptor Ryk, which is expressed in the corticospinal tract, acts as a chemorepulsive receptor for the growth of corticospinal axons, and consequently its inhibition increases axonal growth and improves functional recovery after SCI [36], [38]. Notably, the Ryk-mediated effects on axonal growth may also depend on the presence of Fz receptors [39]. Although the potential roles of Fz receptors in axonal regeneration after CNS injury have not been studied, it should be noted that, under physiological conditions, these receptors are involved in axonal growth and/or function during CNS development [63]–[67] and adulthood [65], [66]. In agreement with these observations, several Fz receptors are expressed in cultured hippocampal neurons in both their cellular soma and neurites [68]. Interestingly, here we show that in the NL spinal cords, Fz7 is expressed in fine and extremely elongated structures with small engrossments, which may resemble axons. Moreover, after SCI, a fine punctate immunolabelling for Fz2 and Fz7 was observed in the damaged parenchyma at early times post-injury, which in both cases disappeared at 14 dpi. Finally, our results have demonstrated that Fz5 is expressed in axons that were present in the damaged nervous tissue at all evaluated times post-SCI, although this receptor was not expressed in axonal projections in the NL spinal cords.

Another crucial process during the progression of SCI is the mobilisation and differentiation of the multipotent stem cells and glial precursors that are present in the spinal cord, to replace those cells that have been lost due to injury progression [69]–[72]. However, most of these cells differentiate into astrocytes but not neurons or oligodendrocytes, thereby enhancing scar formation and possibly worsening SCI outcome [69]. Several studies have supported the modulatory role of the Wnt family of proteins in cell differentiation after a CNS injury, beyond their well-known involvement during CNS development [22], [73], [74]. Specifically, the activation of the Wnt canonical signalling pathway increases the proliferation of cultured neural stem cells subjected to hypoxia [41]. Moreover, Wnt5a over-expression in transplanted stem/progenitor cells in parkinsonian mice increases the generation of dopaminergic neurons [16]. Furthermore, Wnt3a administration after SCI enhances cell differentiation to neurons while reducing cell differentiation to astrocytes [40]. Interestingly, we observed that Fz2, -5, -8, -9 and -10 were expressed in cells belonging to the central canal, where multipotent stem cells can be found. Moreover, from 5 dpi onwards, ramified NG2^+^ glial precursors are mainly located in the lesion epicentre [75], [76] where, at 7 and 14 dpi in this study, almost all detectable Fz receptors were expressed in cells displaying a highly ramified profile. In agreement, our double immunohistochemistry results showed that Fz5 was expressed in NG2+ glial precursors that were located in the damaged areas at all analysed times post-SCI, although Fz5 expression in NG2^+^ glial precursors was more evident at 7 and 14 dpi, correlating with the maximal presence of this cell type in the lesioned nervous tissue.

To further contextualise our results, it is important to note that the functioning of Fz receptors is extremely complex, as their potential roles depend on many aspects, such as their Wnt ligand specificity, their cell-specific expression and sub-cellular location, the presence of the different endogenous Wnt-modulatory molecules, and the activation state of other intracellular signalling cascades that may interact with the different Wnt signalling pathways [8].

In summary, we have demonstrated for the first time that almost all Fz receptors are physiologically expressed in the uninjured adult rat spinal cord and displaying distinct spatial expression patterns, and that Fz5 is expressed in different cell types such as neurons, oligodendrocytes, microglial cells, astrocytes and NG2^+^ glial precursors. Moreover, we also showed that the spatio-temporal expression patterns of Fz receptor mRNA and protein are dramatically altered during the progression of a contusive SCI. Furthermore, our results have demonstrated that the cellular Fz5 expression pattern suffered evident alterations after SCI, being observed in oligodendrocytes, reactive astrocytes and microglia/macrophages, NG2^+^ glial precursors and axonal projections. However, although our results strongly suggest a modulatory action of Fz receptors in the progression of SCI, we cannot draw conclusions about the specific functions of these receptors. Thus, further experimental studies must be performed to shed light on this novel and promising research field.

## Supporting Information

Figure S1 **Evaluation of motor function recovery in the open-field test.** The Basso-Beattie-Bresnahan (BBB) locomotor scale was used to establish a homogeneous group where only those animals with a BBB score between 0 and 3 at day 1 after surgery and with a similar functional improvement up to 14 days post-injury (dpi) were included in the study.
(TIF)
