# Supplementary figures and images for: Spatio-Temporal Expression Pattern of Frizzled Receptors after Contusive Spinal Cord Injury in Adult Rats
Source: PLoS One. 2012 Dec 10;7(12):e50793. doi: 10.1371/journal.pone.0050793 (PMC3519492; doi:10.1371/journal.pone.0050793)

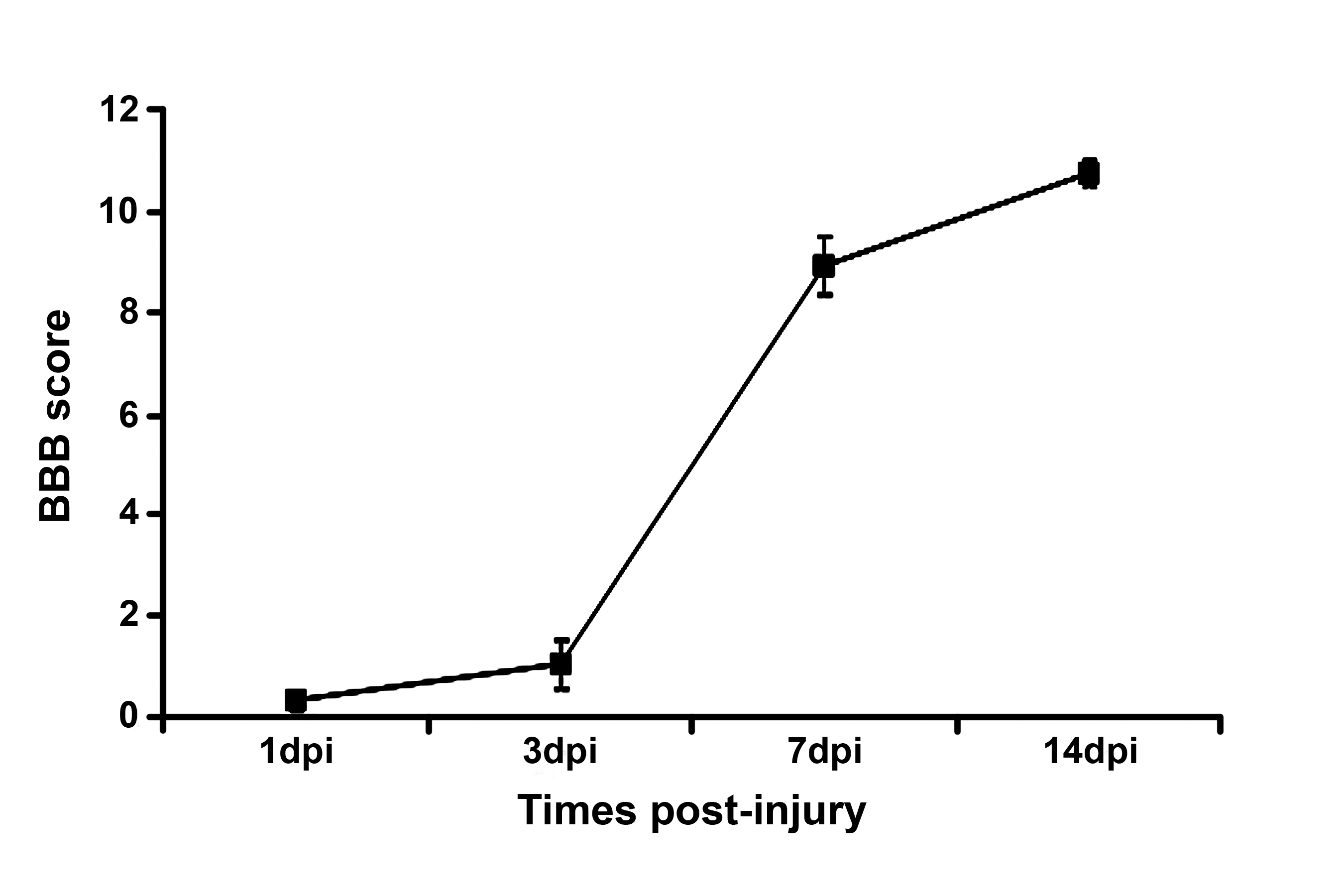

Supplement: Figure S1 — Evaluation of motor function recovery in the open-field test. The Basso-Beattie-Bresnahan (BBB) locomotor scale was used to establish a homogeneous group where only those animals with a BBB score between 0 and 3 at day 1 after surgery and with a similar functional improvement up to 14 days post-injury (dpi) were included in the study. (TIF) [file pone.0050793.s001.tif]
